# Global Phosphoproteomics Analysis of IBRS-2 Cells Infected With Senecavirus A

**DOI:** 10.3389/fmicb.2022.832275

**Published:** 2022-01-26

**Authors:** Jieyi Li, Zhongwang Zhang, Jianliang Lv, Zhongyuan Ma, Li Pan, Yongguang Zhang

**Affiliations:** ^1^State Key Laboratory of Veterinary Etiological Biology, Key Laboratory of Animal Virology of Ministry of Agriculture, Lanzhou Veterinary Research Institute, Chinese Academy of Agricultural Sciences, Lanzhou, China; ^2^Jiangsu Co-Innovation Center for the Prevention and Control of Important Animal Infectious Disease and Zoonose, Yangzhou University, Yangzhou, China; ^3^Lanzhou Institute of Husbandry and Pharmaceutical Sciences, Chinese Academy of Agricultural Sciences, Beijing, China

**Keywords:** protein modification, phosphoproteome, bioinformatics, Senecavirus A, pathway analysis

## Abstract

Phosphorylation is a widespread posttranslational modification that regulates numerous biological processes. Viruses can alter the physiological activities of host cells to promote virus particle replication, and manipulating phosphorylation is one of the mechanisms. Senecavirus A (SVA) is the causative agent of porcine idiopathic vesicular disease. Although numerous studies on SVA have been performed, comprehensive phosphoproteomics analysis of SVA infection is lacking. The present study performed a quantitative mass spectrometry-based phosphoproteomics survey of SVA infection in Instituto Biologico-Rim Suino-2 (IBRS-2) cells. Three parallel experiments were performed, and 4,520 phosphosites were quantified on 2,084 proteins. Gene Ontology (GO) functional enrichment and Kyoto Encyclopedia of Genes and Genomes (KEGG) pathway enrichment analyses showed that many phosphorylated proteins were involved in apoptosis and spliceosome pathways, and subcellular structure localization analysis revealed that more than half were located in the nucleus. Motif analysis of proteins with differentially regulated phosphosites showed that proline, aspartic acid, and glutamic acid were the most abundant residues in the serine motif, while proline and arginine were the most abundant in the threonine motif. Forty phosphosites on 27 proteins were validated by parallel reaction monitoring (PRM) phosphoproteomics, and 30 phosphosites in 21 proteins were verified. Nine proteins with significantly altered phosphosites were further discussed, and eight [SRRM2, CDK13, DDX20, DDX21, BAD, ELAVL1, PDZ-binding kinase (PBK), and STAT3] may play a role in SVA infection. Finally, kinase activity prediction showed 10 kinases’ activity was reversed following SVA infection. It is the first phosphoproteomics analysis of SVA infection of IBRS-2 cells, and the results greatly expand our knowledge of SVA infection. The findings provide a basis for studying the interactions of other picornaviruses and their mammalian host cells.

## Introduction

Senecavirus A (SVA), formerly known as Seneca Valley virus (SVV), is known for its selective tropism toward cancers and belongs to the genus *Senecavirus*, family *Picornaviridae*. Like other picornaviruses, SVA is a single-stranded, positive-sense, non-enveloped virus (Hales et al., 2008). The first reported SVA, named SVV-001 in 2002, was identified incidentally as a contaminant in cultured PER.C6 cells (Adams et al., 2015). Since 2014, outbreaks of SVA-associated vesicular diseases in pigs have occurred in many countries worldwide (Zhu et al., 2017b; Saeng-Chuto et al., 2018). The first report of SVA infection in China was in 2015 (Zhu et al., 2017b). Other important members of *Picornaviridae*, such as foot-and-mouth disease virus (FMDV) and swine vesicular disease virus, have received much attention, especially their pathogenic mechanisms (Fu et al., 2019) and detection and prevention technologies (Senthilkumaran et al., 2017; Poonsuk et al., 2018). Although SVA is advancing through clinical trials due to its oncolytic and detection properties (Reddy et al., 2007; Zhang et al., 2019), little is known about how host cells respond to SVA infection. As an emerging infectious disease in pigs, the potential impact of SVA could be significant, and research is needed.

Proteins are regulated by post-translational modification (PTM) in all organisms. Covalent connections between functional groups and amino acid residues can greatly expand the functions of proteins. Up to 30% of proteins are phosphorylated on at least one amino acid residue (Pinna and Ruzzene, 1996), and nearly 2% of the eukaryotic genome contains genes encoding protein kinases (Caenepeel et al., 2004). In mammals, protein phosphorylation mainly occurs on serine, threonine, and tyrosine residues, and kinases and phosphatases, respectively, regulate the reversible cycling of protein phosphorylation and dephosphorylation. Numerous studies have shown that phosphorylation plays a vital role in cell signal transduction, regulating the assembly and activation of the inflammasome (Gong et al., 2018), apoptosis (Shiladitya et al., 2017), the proliferation of tumor cells (Huang et al., 2019; Seo et al., 2019), and interactions between viruses and host cells (Öhman et al., 2015). Protein phosphorylation is also widespread in bacteria and parasites (Esser et al., 2016; Wang et al., 2019).

Protein phosphorylation in host cells occurs in response to viral infection and helps to regulate complex signaling networks, as demonstrated for HIV (Wojcechowskyj et al., 2013), Influenza C virus (Goto et al., 2017), Hepatitis C virus (Goonawardane et al., 2017), and Ebola virus (Biedenkopf et al., 2016). However, global protein phosphorylation responses of host cells infected with SVA remain unknown. As an etiologic agent of pigs, SVA can infect and replicate in many porcine cell lines, previous study showed the Instituto Biologico-Rim Suino-2 (IBRS-2) cell line was most permissible to SVA infection (Zhu et al., 2017b; Zhang et al., 2021). Therefore, we characterized protein phosphorylation in IBRS-2 cells using liquid chromatography-tandem mass spectrometry (LC-MS/MS) following infection with SVA. Three parallel experiments were performed to ensure the repeatability and accuracy of the data. The study identified 9,034 phosphosites on 2,794 proteins and obtained quantitative information for 4,520 sites on 2,084 proteins. Sixty-five upregulated, and 180 downregulated sites (>1.5-fold) were subjected to bioinformatic analysis. The results lay the foundation for future investigation of phosphorylated proteins in IBRS-2 cells infected with SVA. Furthermore, the findings could be applied to study other mammalian cell activities related to phosphorylation.

## Materials and Methods

### Cell Lines and Viruses

IBRS-2 cells (ATCC) were cultured in Dulbecco’s modified Eagle’s medium (DMEM; Gibco, Grand Island, NY, United States) supplemented with 10% fetal bovine serum (Gibco) and 1% penicillin–streptomycin–gentamicin solution (Solarbio, Beijing, China) at 37°C with 5% CO_2_ in 75 cm^2^ cell culture flasks. SVA strain A/ZJ/2015 was isolated and preserved at the OIE/National Foot-and-Mouth Disease Reference Laboratory (Lanzhou, Gansu, China).

### Infection

IBRS-2 cells were seeded in 75 cm^2^ cell culture flasks until they reached 80% confluence. The growth medium was removed, and cells were washed three times with sterile phosphate-buffered saline (PBS; pH 7.2; Gibco). Cells were infected with 3.5 ml of SVA strain A/ZJ/2015 at a multiplicity of infection (MOI) of 0.5 and incubated for 2 h at 37°C with 5% CO_2_ (Zhang et al., 2021). A 6.5 ml volume of DMEM was then added to culture flasks, and culturing was continued until significant cytopathic effects (CPEs) were evident (9 h). Uninfected cells were incubated in DMEM alone as a non-infected control (NC) group. IBRS-2 cells infected with 0.5 SVA MOI for 9 h served as samples for LC-MS-MS analysis.

### Indirect Immunofluorescence

IBRS-2 cells were infected with SVA strain A/ZJ/2015 (MOI = 0.5) when they reached 70% confluence. Cells were inoculated at 37°C with 5% CO_2_ over a 1-h period, the medium was replaced with fresh DMEM, and continued incubation. DMEM was removed at 0, 6, 9, and 12 hpi, and cells were fixed with cold methanol for 10 min. Samples were washed with PBS, blocked with 1% bovine serum albumin (Solarbio) for 30 min, and SVA proteins were detected using rabbit polyclonal anti-SVA-VP1 primary antibody (prepared and stored in our laboratory) and Dylight 488-conjugated goat anti-rabbit IgG secondary antibody (Abbkine, Wuhan, China). Cell nuclei were stained for 20 min with 10 μg/ml Hoechst 33342 (Solarbio), and immunofluorescence microscopy images were captured using a confocal laser scanning microscope (Leica, Wetzlar, Germany).

### Protein Extraction

Cells were resuspended in lysis buffer (8 M urea, 1% protease inhibitor, 1% phosphatase inhibitor, 3 μM Trichostatin A, and 50 mM Nicotinamide) and sonicated three times on ice using a high-intensity ultrasonicator, and precipitates were removed by centrifugation at 12,000*g* for 10 min at 4°C. According to the manufacturer’s instructions, the supernatant was collected, and the protein concentration was determined with a BCA kit (Beyotime, Shanghai, China).

### Trypsin Digestion

The same amount of cell protein from NC and treatment groups was digested with trypsin (Promega, Wisconsin, United States). An appropriate amount of standard protein (prokaryotic expressed protein) was added based on the two samples’ volume to control the entire enzymatic digestion process. Trichloroacetic acid was added to a final concentration of 20%, and samples were mixed by vortexing and allowed to settle for 2 h. The precipitate was collected by centrifugation at 4,500*g* for 5 min and washed with cold acetone twice. Triethylammonium bicarbonate (Sigma, Darmstadt, Germany) was added to a final concentration of 200 mM, and samples were sonicated and treated with trypsin (1:50 w/w) overnight. Next, 5 mM dithiothreitol (Sigma) was added to reduce the protein solution for 30 min at 56°C, and samples were incubated with 11 mM iodoacetamide (Sigma) in the dark for 15 min.

### Affinity Enrichment

Peptide mixtures were first incubated with IMAC microspheres suspension with vibration in enrichment buffer (1 ml, 50% acetonitrile/6% trifluoroacetic acid) and incubated overnight at 4°C with gentle shaking. The IMAC microspheres with enriched phosphopeptides were collected by centrifugation, and the supernatant was removed. The IMAC microspheres were washed with 1 ml 50% acetonitrile/6% trifluoroacetic acid and 1 ml 30% acetonitrile/0.1% trifluoroacetic acid sequentially to remove nonspecifically adsorbed peptides. Finally, peptides were eluted with 10% ammonia and cleaned with C18 ZipTips according to the manufacturer’s instructions.

### Liquid Chromatography-Tandem Mass Spectrometry

The enriched phosphorylated peptides were dissolved in 0.1% formic acid (solvent A), then loaded onto a reversed-phase analytical column [40 μl resin (50% slurry), PTM-Bio, PTM-402] attached to a Nano Elute UPLC system at a constant flow rate of 700 nl/min. The gradient employed for elution was 2–22% solvent B (0.1% formic acid in 98% acetonitrile) over 50 min, increased to 35% solvent B over 2 min, then to 90% over 3, and holding at 90% for 5 min. Peptides were subjected to capillary ionization, followed by trapped ion mobility time of flight spectroscopy. The electrospray voltage applied was 1.6 kV, the *m*/*z* scan range was 100–1,700 for full scans, and the parallel cumulative serial fragmentation (PASEF) data acquisition mode was employed. Ten primary and secondary mass spectra were collected sequentially (precursor ions ranged from 0 to 5) in PASEF mode. The dynamic exclusion time of MS/MS scans was set to 24 s to avoid repeated scanning of precursor ions.

### Parallel Reaction Monitoring of Phosphorylated Proteins

The tryptic peptides were dissolved in 0.1% formic acid (solvent A) and isolated with an EASY-nLC 1000 UPLC system. The gradient applied for elution was an increase from 1 to 18% solvent B (0.1% formic acid in 90% acetonitrile) over 38 min, followed by 18–32% over 4 min, which was then increased to 80% over 4 min, and held at 80% for 4 min. The flow rate was maintained at 450 nl/min.

Phosphorylated peptides were isolated using a UPLC system, then loaded onto an NSI source and analyzed by MS/MS using a Q ExactiveTM HF-X instrument. The electrospray voltage was set to 2.2 kV, and the *m*/*z* range was 510–1,120. Intact peptides were detected using an Orbitrap at a resolution of 120,000 for primary mass spectra and 30,000 for MS/MS spectra. Data-independent scanning was employed as the data acquisition mode. The automatic gain control was 3E6 for full MS scans and 1E5 for MS/MS scans. The maximum IT was set at 140 ms, and the isolation window was set at 1.4 *m*/*z*.

### Database Searching

Secondary MS data were processed with MaxQuant (v1.6.6.0), and tandem mass spectra were searched against the Sus_scrofa_9823_PR_20191031 database (40,701 sequences) concatenated with the reverse-decoy database. The common pollution bank was added to eliminate the impact of contaminating proteins. Trypsin/P was specified as the cleavage enzyme; up to two missed cleavages were allowed, the maximum number of modifications per peptide was five, and the minimum length of peptides was seven amino acids. The mass tolerance for fragment ions in the first search and the main search was set to 20 ppm, while the mass tolerance for secondary fragment ions was set to 0.02 Da. False discovery rate, PSM identification, and protein identification were adjusted to <1%. Cysteine alkylation was set as a fixed modification, and variable modifications included methionine oxidation, acetylation of the protein N-terminus, and phosphorylation of serine, threonine, and tyrosine. All the other parameters in MaxQuant were set to default values.

A label-free quantification algorithm, LFQ, was used for protein quantitation for the proteomic data. For the phosphoproteomic data, the intensities of the phosphosites of all samples were extracted from the MaxQuant software. The confidently identified phosphosites (1% FDR and 75% localization probability) were applied with the mean-centering correction to adjust for sample-specific biases. Phosphosites quantification was divided by protein quantification to remove the effect of protein expression on modification abundance. The Student’s *t*-test examined whether phosphosites were differentially modified between NC and SVA. Quantifications of a given phosphosite in at least two out of three replicates were analyzed by the Student’s *t*-test. While *p* < 0.05 and fold change >1.2 were used to identify differentially modified phosphosites. PCA and Pearson’s correlation analysis was used to evaluate the quantitative stability of biological replicates to measure the reliability of the quantification between biological replicates.

For the parallel reaction monitoring (PRM) database search, peptide settings were as follows: the enzyme was Trypsin [KR/P], the maximum number of missed cleavages was two, the peptide length was 7–35 amino acids, cysteine alkylation was set as a fixed modification, and serine/threonine/tyrosine phosphorylation was set as a variable modification. Regarding the transition settings, precursor charges were set as 2, 3, and 4, ion charges were set as *b* and *y* (corresponding to phosphorylation neutral loss generated when the library is introduced), fragment ions were set from ion 3 to the last ion, and the ion match tolerance was set as 0.02 Da. The data processing method of PRM is the same as that of phosphoproteomic.

### Bioinformatics Analysis

Subcellular location prediction of phosphorylated proteins was performed using Wolfpsort, Soft MoMo (motif-x algorithm) to analyze the models of sequences constituted with amino acids in specific positions of modified 13-mers for phosphorylation (six amino acids upstream and downstream of the site). The protein sequence database was derived from Sus scrofa of the Uniprot database (version: 20200506, sequences: 49,793), and all other parameters were set to default values.

Gene Ontology (GO) annotation was performed against the UniProt-GOA database. All proteins of this species were used as background. Firstly, identified proteins IDs were switched to UniProt IDs and mapped to GO IDs. InterProScan was employed to predict protein function based on the protein sequence alignment method when the UniProt-GOA database did not annotate proteins. Proteins were classified according to their cellular components, molecular functions, and physiological processes. Protein domain annotation was performed by InterProScan software and the InterProScan domain database. Similarly, Kyoto Encyclopedia of Genes and Genomes (KEGG) pathway annotation was performed against the online KEGG Automatic Annotation Server, and the annotation results were mapped to the KEGG pathway database using KEGG mapper. Benjamini-Hochberg (BH) method was used to correct the *p* value.

A two-tailed Fisher’s exact test was employed to assess the enrichment of proteins with differentially regulated phosphosites (GO, KEGG pathway, and protein domain; *p* < 0.05 was considered significant).

Protein–protein interaction (PPI) analysis was performed using the Neighboring Gene (STRING) database. All differentially regulated proteins were searched against this database, those with an interaction confidence score ≥0.7 were retained, and all other parameters were set as default values. The PPI network was visualized using Cytoscape. Kinase-substrate interactions were identified by PPI analysis. Kinases can regulate multiple substrates, and one phosphosite may be regulated by more than one kinase. The differentially regulated ratio threshold was set as 1.5, the significant change threshold was 0.05, and the predicted kinases and significantly altered phosphosites were chosen to construct a kinase-substrate regulatory network. Skyline software was used for peak picking and determination of peak area ratios, and the Student’s *t*-test method was used to calculate the *p* value of PRM.

### Prediction of Kinase-Substrate Regulation and Kinase Activity

The prediction of kinase-substrate regulation was conducted using GPS5.0 software, based on the theory of short linear motifs (SLMs) around phosphorylation sites (p-sites), and a “medium” threshold was chosen. The corresponding kinases were predicted by comparing them with kinase sequences in the IEKPD2.0 database. PPI information was used to remove potentially false-positive hits.

Regarding kinase activity, the activity state is reflected in the phosphorylation level of the substrates. The normalized enrichment score (NES) of enrichment results served as the kinase activity score. Kinases for which the phosphorylation of substrates was increased were considered positive, and vice versa.

### Western Blot

The IBRS-2 cells were placed at a 60 mm dish, then infected with SVA when they reached 80% confluence. Whole cellular lysates (30 μg protein) were subjected to immunoblot analysis with antibodies against STAT3, β-actin, phosphorylated-STAT3-Ser727, CDK2 (Abcam), and SVA-VP1 (self-made). Secondary antibodies were horseradish peroxidase-coupled anti-rabbit IgG antibodies (Thermo). Protein-bound antibodies were visualized with ECL plus detection reagents (Thermo). The protein expression was detected by ImageJ software and represented by area value.

### CDK2 Kinase Activity Analysis

The activity of porcine CDK2 was tested by a double antibody sandwich enzyme-link immunoassay Kit (Jiangsu Kete Biotechnology). The standard sample 50 μl and tested sample 50 μl (10 μl cell lysates were diluted by 40 μl sample diluent) were added to the ELISA plate, then sealing the plate and incubate at 37°C for 30 min. The solution was discarded in an ELISA plate and washed five times by wash buffer. Around 50 μl enzyme were added to each well except for the negative control followed by incubating and washing the plate as mentioned above. About 50 μl chromogenic fluid A and B were added and mixed, then incubated at 37°C for 30 min. About 50 μl terminated buffer was added to each well, and the OD value of each well was detected at 450 nm. The OD value is positively correlated with enzyme activity.

## Results

### Virus Infection and Determination of Optimal Collection Time

**Figure 1:**
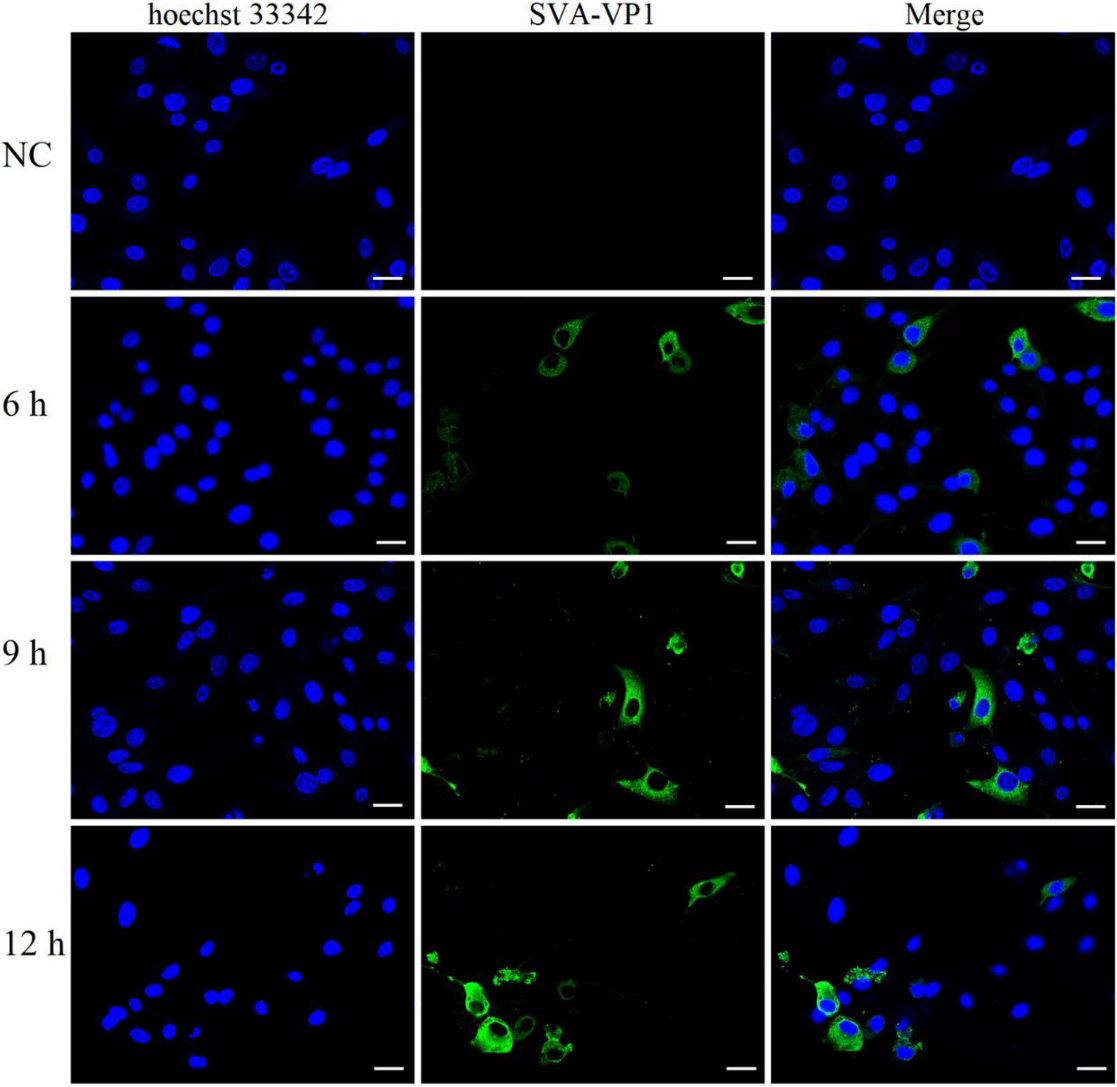
Indirect immunofluorescence analysis of IBRS-2 cells infected with Senecavirus A (SVA). The cells were detected for indirect immunofluorescence assays with the rabbit anti-SVA VP1 antibodies (green), and nuclei were counterstained with 10 μg/ml hoechst33342 (blue). Fluorescent images were acquired with a confocal laser scanning microscope. Bright field, Scale bar = 25 μm.

Indirect immunofluorescence was measured to determine the optimal SVA infection time for collecting cells for proteomics and phosphoproteomics experiments to obtain comprehensive SVA infection data. SVA-VP1 was located in the cytoplasm, consistent with the picornavirus replication mechanism (Figure 1). SVA can be detected in cells after 6 hpi. Around 9 hpi was chosen as the optimal time for SVA infection of IBRS-2 cells for the number of infected cells increased after 6 hpi, and many infected cells dead at 12 hpi. Cells collected at this time point were subjected to proteomics and phosphoproteomics analyses by high-resolution LC-MS/MS.

### Global Detection of Phosphosites in IBRS-2 Cells Infected With SVA

**Figure 2:**
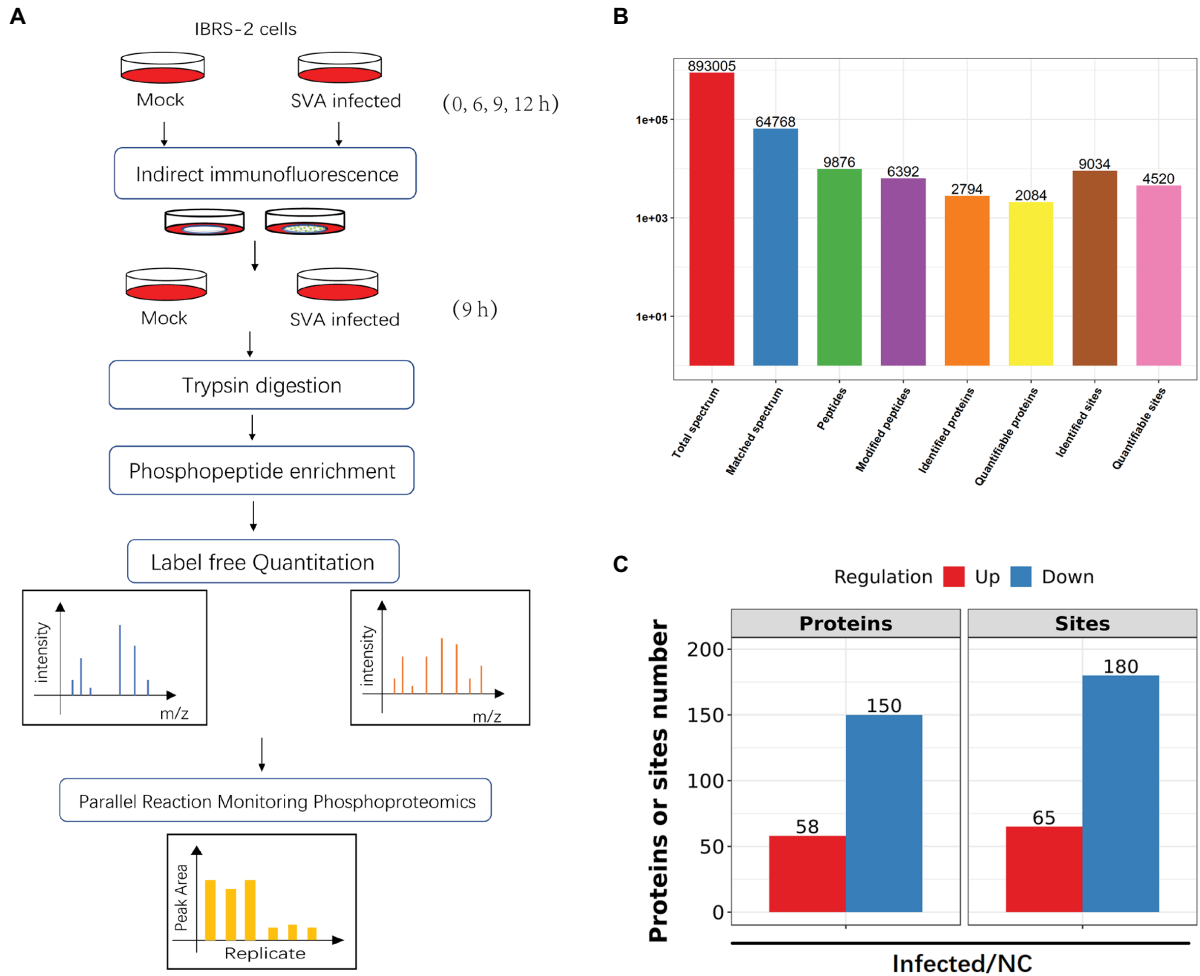
A quantitative overview of the phosphoproteomic analysis of phosphoproteins with differentially regulated phosphosites. **(A)** Schematic workflow of phosphoproteomic analysis of IBRS-2 cells infected with SVA. **(B)** Overview of phosphoproteomics analysis, including identifying and quantifying phosphoproteins and phosphopeptides. **(C)** The significant change proteins after SVA infection upon proteomics analysis; the number of phosphoproteins and phosphosites based on a ratio of infected vs. non-infected control (NC) groups >1.5 (*p* < 0.05) for the upregulated threshold and <1.5 (*p* < 0.05) for the downregulated threshold.

Global phosphorylation analyses were performed in biological triplicate on SVA-infected IBRS-2 cells using affinity enrichment, followed by high-resolution LC-MS/MS (Figure 2A). Proteomics analysis of samples was performed to investigate whether protein expression levels can affect the level of phosphorylation. To eliminate the effect of protein expression levels, we conducted a proteomics analysis before the phosphoproteomics analysis and normalized the data (Supplementary Table S1).

The phosphoproteomics study identified 4,520 phosphosites on 2,084 proteins with quantitative information (Figure 2B). A change in phosphorylation by ≥1.5-fold compared with uninfected cells was defined as the threshold for significant upregulation, and the threshold for significant downregulation was ≤1.5-fold. Using these parameters, we detected 65 upregulated sites on 58 proteins and 180 downregulated sites on 150 proteins (Figure 2C; Supplementary Table S2).

The only protein modified at more than three residues was SRRM2, which was modified at seven residues (six of which were serine). SRRM2 is a Ser-Arg-rich (SR) protein that contains two characteristic arginine-serine (RS) domains. SR proteins are central regulators of cellular splicing and can be modulated by phosphorylation of the RS domains (Long and Caceres, 2009), and the relevance between SRRM2 and SVA has not been discussed. Asparagine synthetase (ASNS) was the most differentially phosphorylated protein (upregulated 4.486-fold). ASNS convert aspartate to asparagine, which is accompanied by glutamine deamidation. Previous studies on ASNS have focused on therapeutic strategies for cancers, but the relevance of ASNS to viral infection has not been reported previously.

### Analysis of Phosphorylated Peptide Motifs

**Figure 3:**
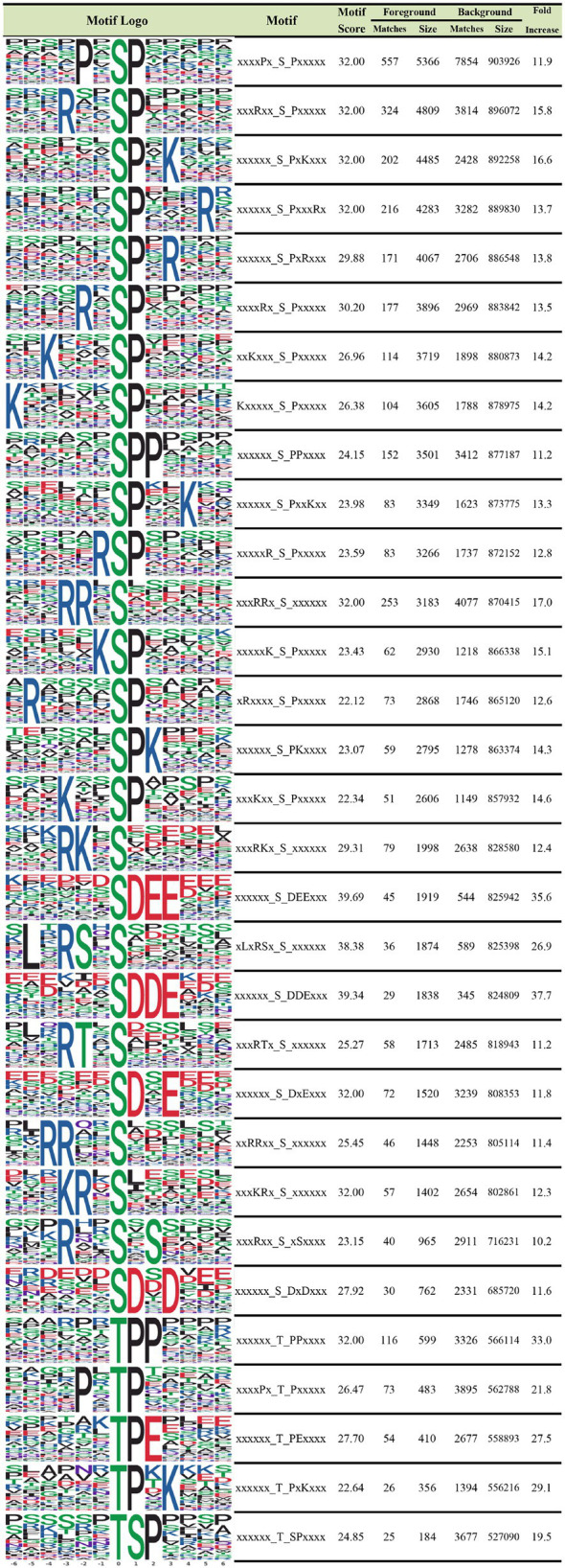
Motif analysis of the identified phosphoproteins in IBRS-2 cells infected with SVA. The obtained phosphosites’ characteristics at serine and threonine residues and their enrichment statistics are shown, and serine and threonine motifs with a fold increase >10 are included.

To further examine the properties of phosphorylated sites, we explored the regulation of flanking sequences from six amino acids upstream to six amino acids downstream of the modified sites. This approach was employed to reveal the sequence characteristics of phosphosites and identify enzymes associated with phosphorylation. We identified 26 serine motifs and five threonine motifs that were increased more than 10-fold (Figure 3). According to our analysis, a P residue at the +1 position was the most frequent in both serine and threonine motifs. In serine motifs with a D residue at the +1 to +2 positions and an E residue at the +2 to +3 positions, the increase was >30-fold, and the motif scores were up to 39.69. Regarding the threonine motif, a P residue at the +1 to +2 position and an E residue at the +2 position was most frequently observed upstream of the motifs. P residues in threonine motifs at the +1 and + 2 positions were increased up to 33-fold. Meanwhile, R residues were most abundant at the −3 and −1 positions and P residues at the −2 position downstream of threonine motifs. Notably, D and E are both negatively charged, and both were most abundant in the serine motif, while R is positively charged and was most abundant upstream of threonine residues. In conclusion, kinases and phosphatases identified in this study prefer to bind to serine motifs with P residues at +1, D residues at +1 to +2, and E residues at +2 to +3 positions, as well as threonine motifs with P residues at +1, E residues at +2, and R residues at −2 positions.

### Protein Annotation and Functional Classification of Identified Phosphosites

To further explore the functions and characteristics of proteins with identified phosphosites, GO, protein domain, KEGG pathway, and subcellular localization, bioinformatics analyses were performed for annotation. Fisher’s exact test method used KEGG pathway enrichment analysis on proteins with detected phosphosites.

**Figure 4:**
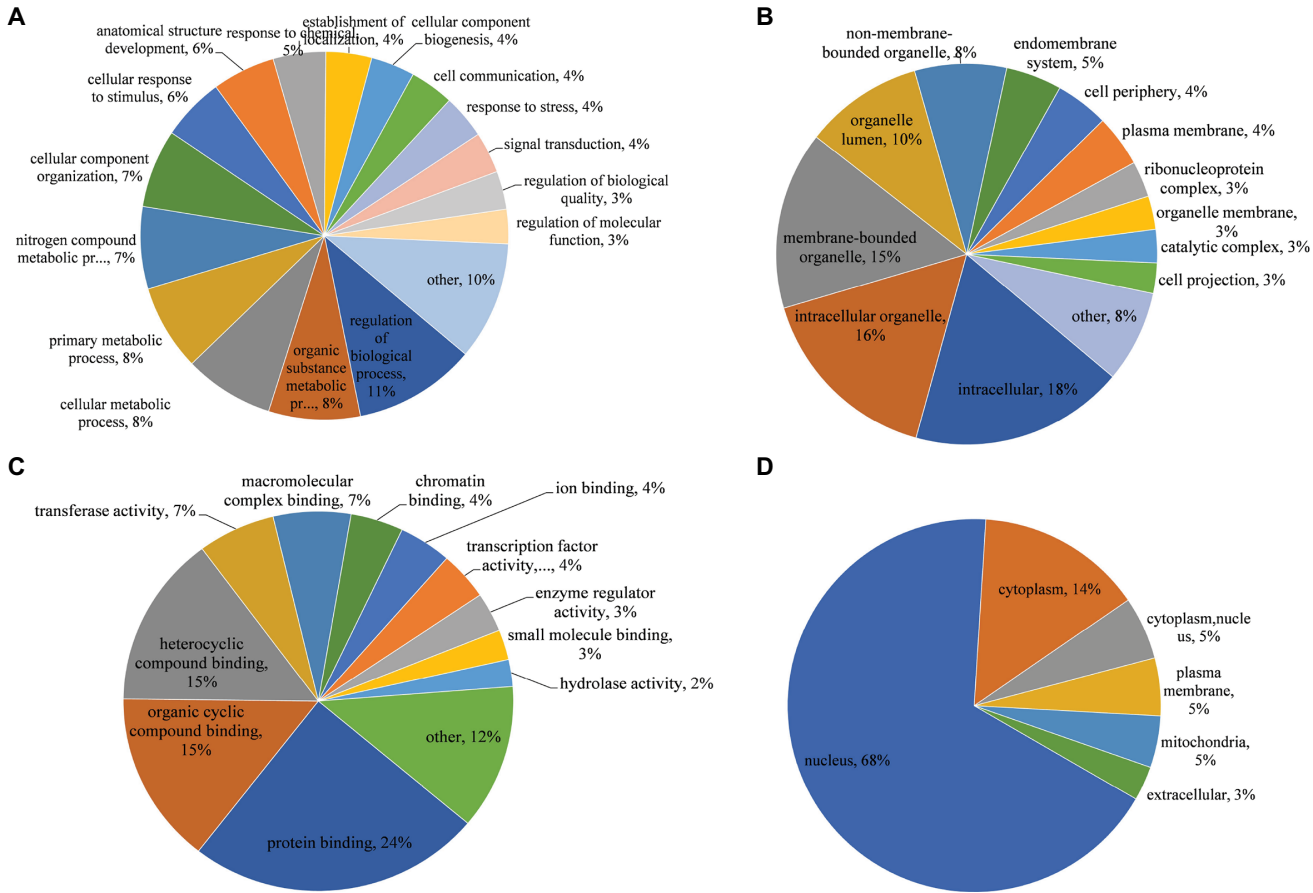
Gene Ontology (GO) functional annotation and subcellular location of phosphorylated proteins in IBRS-2 cells infected with SVA. **(A)** Biological process. **(B)** Cellular component. **(C)** Molecular function. **(D)** Subcellular location.

In the GO biological process category, regulation of the biological process, organic substance metabolic process, cellular metabolic process, and primary metabolic process were the most prominent terms, accounting for 11, 8, 8, and 8%, respectively (Figure 4A). The top three classes in the cellular component category were intracellular (18%), intracellular organelle (16%), and membrane-bounded organelle (15%; Figure 4B). In the molecular function category, the top three classes were protein binding (24%), organic cyclic compound binding (15%), and heterocyclic compound binding (15%), implying an essential role for phosphorylation in molecular binding, especially protein binding (Figure 4C). In the subcellular localization category, 68 and 14% of proteins were located in the nucleus and cytoplasm, respectively, suggesting that most phosphorylation events occurred in these locations. Furthermore, 5% of proteins with differentially regulated phosphosites were located in mitochondria, suggesting that mitochondrial proteins may play an important role in regulating SVA infection (Figure 4D).

### Functional Enrichment of Proteins With Differentially Regulated Phosphosites

**Figure 5:**
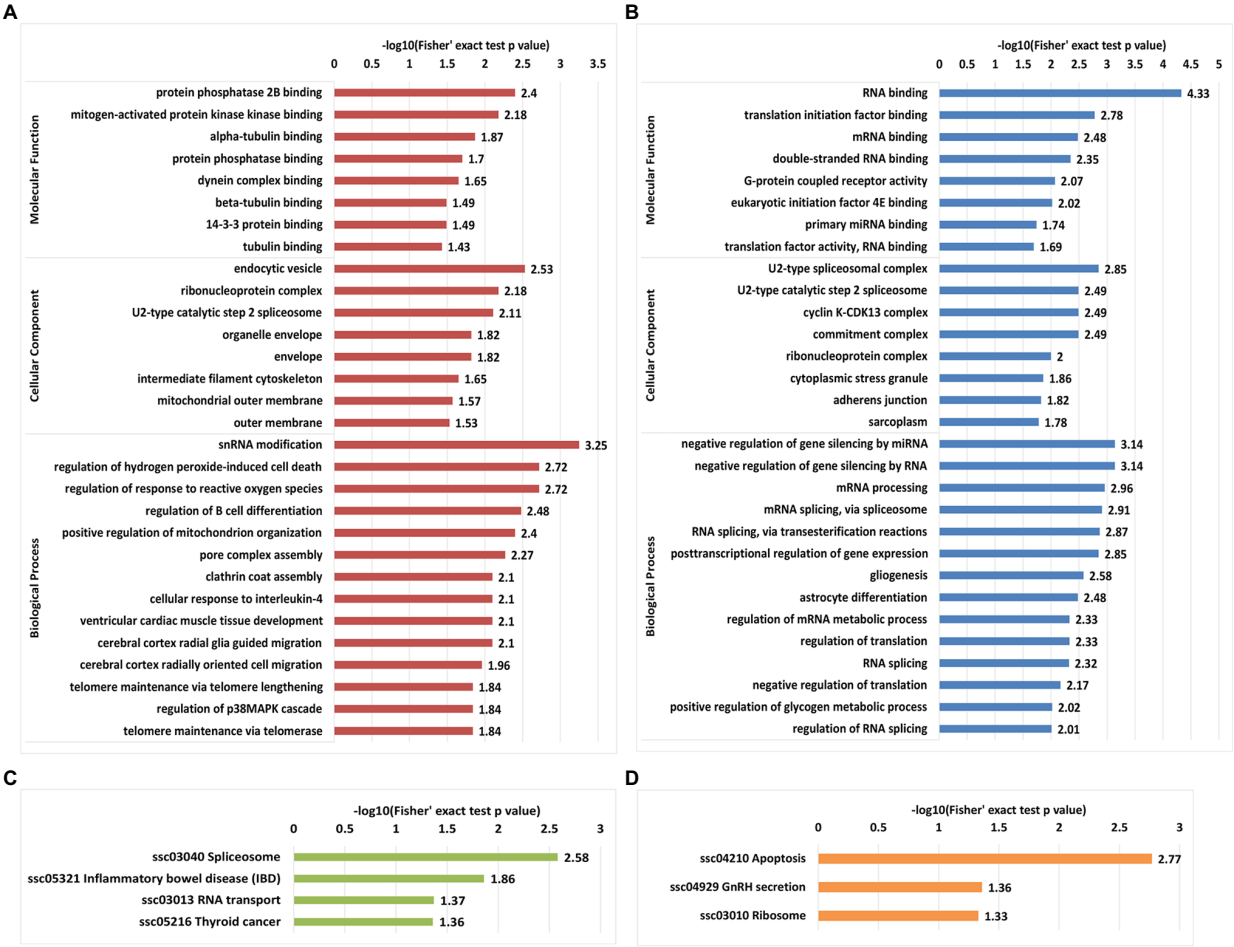
Gene Ontology functional enrichment and Kyoto Encyclopedia of Genes and Genomes (KEGG) pathways enrichment analyses of proteins with differentially regulated phosphosites. **(A)** GO-based enrichment analysis of proteins with upregulated phosphosites. **(B)** GO-based enrichment analysis of proteins with downregulated phosphosites. **(C)** KEGG pathway enrichment analysis of proteins with upregulated phosphosite. **(D)** KEGG pathway enrichment analysis of proteins with downregulated phosphosites.

Gene Ontology enrichment and KEGG enrichment analyses were performed to predict the biological functions of proteins with differentially regulated phosphosites in IBRS-2 cells following SVA infection. Regarding proteins with upregulated phosphosites, protein phosphatase 2B binding, and mitogen-activated protein kinase binding were the most enriched terms among the molecular function GO category, which is a foregone result that the MAPK pathway is regulated by consecutive phosphorylation of its substrate. An endocytic vesicle, ribonucleoprotein complex, and U2-type catalytic step 2 spliceosome terms were enriched in the cellular component category. In the biological process category, snRNA modification, regulation of hydrogen peroxide-induced cell death, and response to reactive oxygen species were the top three subcategories, consistent with the annotation results showing that 5% of proteins with upregulated proteins are upregulated phosphorylated were located in mitochondria (Figure 5A).

Unlike the upregulated phosphosites, the enrichment results for proteins with downregulated phosphosites yielded distinct characteristics for two GO categories (molecular function and biological process). RNA binding was the most significantly enriched term in the molecular function category, followed by translation initiation factor binding and mRNA binding. In the biological process category, the top five processes were the gene expression process, including negative regulation of gene silencing by miRNA, negative regulation of gene silencing by RNA, mRNA processing, and mRNA splicing spliceosome and RNA splicing *via* transesterification reactions. Consistent with the above results, evaluation of the cellular component category revealed that the top four components were U2-type spliceosomal complex, U2-type catalytic step 2 spliceosome, cyclin K-CDK13 complex, and the commitment complex, indicating that downregulated phosphosites in IBRS-2 cells infected with may tend to regulate the spliceosome and the cell cycle (Figure 5B).

Kyoto Encyclopedia of Genes and Genomes pathway enrichment analysis was performed on up- and downregulated phosphosites to investigate the metabolic processes and signaling pathways in which the differentially phosphorylated proteins are involved in IBRS-2 cells in response to SVA infection. Proteins with upregulated phosphosites were markedly enriched in apoptosis pathways, while for proteins with downregulated phosphosites, spliceosome pathways were the most enriched, followed by those related to inflammatory bowel disease and RNA transport (Figures 5C,D). Overall, the functional enrichment results are consistent with the findings of previous studies on liver cells of mice and Vero E6 cells infected with SARS-CoV-2 (Bouhaddou et al., 2020; Zhang et al., 2020).

### Protein–Protein Interaction Network Analysis of Proteins With Differentially Regulated Phosphosites

**Figure 6:**
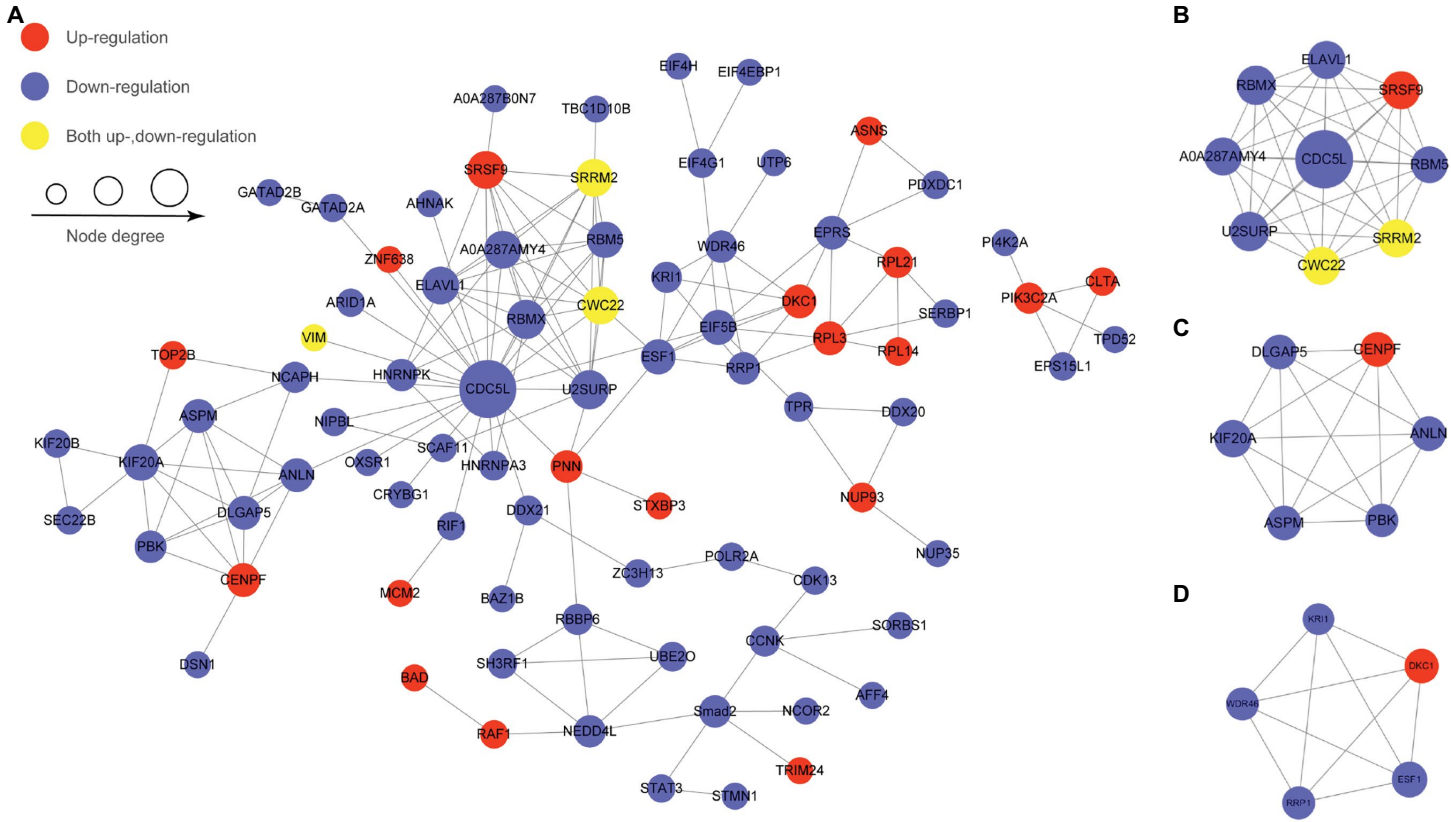
Protein–protein interaction networks of differentially regulated phosphoproteins. Nodes represent the phosphorylated proteins, and edges represent interactions between phosphorylated proteins. The size of nodes represents the combined interaction scores. **(A)** The top 50 most closely interacting proteins interaction network. **(B)** MCODE cluster for spliceosome processes. **(C)** MCODE cluster for mitotic processes. **(D)** MCODE cluster for ribosome synthesis.

Protein–protein interaction analysis was performed by searching for PPI information using the STRING database, and proteins with a confidence score > 0.7 were selected and visualized by Cytoscape software to identify the major biological processes affected by SVA (Figure 6A). CDC5L is the central protein in this PPI network and is linked to most proteins with differentially regulated phosphosites in various pathways, including translation initiation, spliceosome, cell cycle, apoptosis, and ubiquitin-proteasome. MCODE cluster analyses showed that most proteins with differentially regulated phosphosites were involved in spliceosome processes, mitotic processes, and ribosome synthesis (Figures 6B–D). Based on our PPI results, it appears that SVA may employ many ways to regulate biological processes in host cells to support its replication, such as arresting the cell cycle, inhibiting apoptosis, and altering splicing.

### Kinase Analysis of Proteins With Differentially Regulated Phosphosites

**Figure 7:**
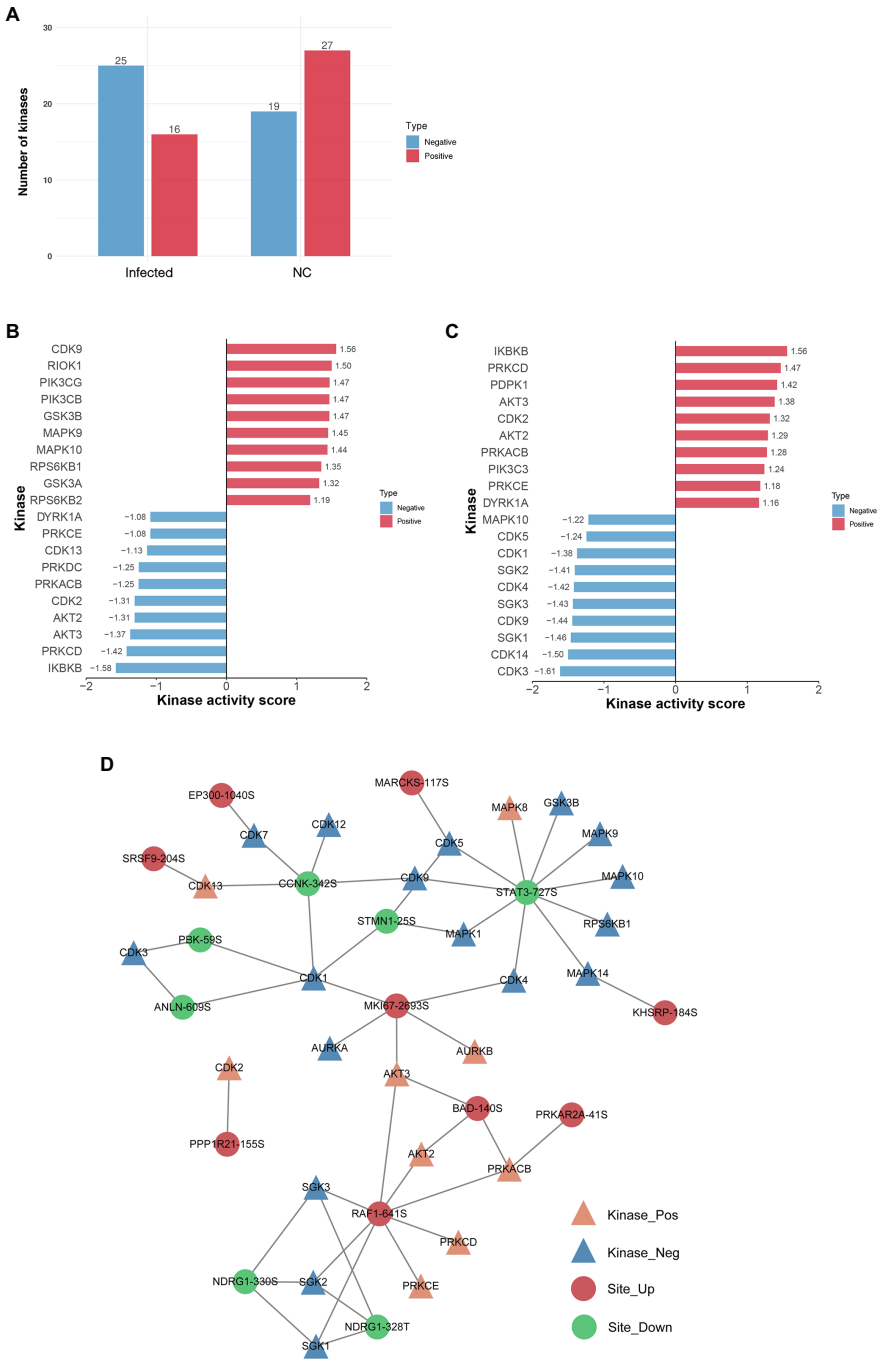
The kinase activity of proteins with differentially regulated phosphosites. **(A)** Statistics of phosphokinase sample. The horizontal axis is the name of the sample, and the vertical axis is the number of kinases. The “Positive” indicates that the kinase activity tends to be activated, and the “Negative” indicates that the kinase activity tends to be inhibited. **(B)** Predictive analysis of phosphokinase activity in the NC group. The *X*-axis represents the kinase activity score, and the *Y*-axis is the activation or inhibition status of the kinase with the top 10 activity scores. “Positive” represents the activation status, and “Negative” represents the inhibition status. **(C)** Predictive analysis of phosphokinase activity in the infected group. **(D)** Phosphokinase-substrate regulatory network. Kinases with significantly activated (NES > 1) or inhibited activity (NES < −1) and phosphorylation sites with significant differences in expression levels (FC > 1.5, value of *p* < 0.05) to construct a kinase regulatory network. Orange represents activated kinase, blue represents inhibited kinase, red represents differentially upregulated phosphosites, and green represents differentially downregulated phosphosites.

Changes in kinase activity can reflect the physiological activity state of host cells. Hence, kinase activity profiling can illuminate the responses of SVA-infected host cells. We predicted the regulatory relationships between differentially regulated phosphoproteins and upstream phosphorylation kinases based on sequence similarity for the phosphosites identified herein. Using GPS software prediction and searching against the iEKPD2.0 database, we identified 1,288 regulatory relationships between these predicted kinases and 496 phosphosites on 259 proteins (Supplementary Table S3). Furthermore, 165 protein kinases belonged to various kinase families, such as CDK, MAPK, CAMKL, PIKK, SGK, GSK, AKT, PKA, PKG, and PKC. Regarding kinase activity, 16 positive and 25 negative kinases were predicted in the infected group, while 27 positive kinases and 19 negative kinases were predicted in the NC group (Figure 7A).

GSEA method predictions of kinase activity and comparison of groups. The NES value obtained by the enrichment analysis was the kinase activity score. A kinase activity score > 0 represents activation, and a kinase activity score < 0 represents inhibition. Interestingly, several kinase activities were reversed following SVA infection. For example, CDK9 and MAPK10 were positive kinases in the NC group but negative in the infected group. Similarly, DYRK1A, PRKCE, PRKACB, PRKCD, AKT2, AKT3, CDK2, and inhibitor of nuclear factor kappa-B kinase subunit beta (IKBKB) were negative kinases in the NC group but positive kinases in the infected group (Figures 7B,C). Interestingly, IKBKB was the most active in the infected group and the most inactive kinase in the NC group.

Finally, based on the regulatory relationships between kinases and phosphosites, a kinase regulatory network was constructed for each comparison group by selecting kinases with significant activation or inhibition and phosphosites with significant differences (Figure 7D). A total of 26 kinases and 16 phosphosites are included in this network. RAF1 and STAT3 are the most prominent potential regulatory kinases, respectively regulating eight kinases (five of which are positive kinases) and 10 kinases (one of which is a positive kinase). These results may tentatively explain the changes in phosphorylation of the identified proteins.

### PRM Validation of Proteins With Differentially Regulated Phosphosites

Forty phosphosites of 27 candidate proteins were selected for PRM analysis to validate the result of phosphoproteomics. Limited by the characteristics of peptides and the abundance of proteins, 30 phosphosites of 21 proteins were quantified (Supplementary Table S4). PRM data were quantified by the peak area of detected phosphorylated peptides. Changes in phosphorylation were quantified by calculating the ratio of the average peak area from three parallel experiments. Excluding sites inconsistent with the results of phosphoproteomics, further analysis was performed by setting the peak area ratio of the infected vs. the NC group as >1.5 or <0.5 as thresholds for significant change resulting in nine proteins with notable changes in phosphorylation [ASNS, ELAVL1, SRRM2, BAD, CDK13, STAT3, DDX21, DDX20, and PDZ-binding kinase (PBK); Table 1].

**Table 1:** Parallel reaction monitoring (PRM) analysis and comparison of the quantitative results for candidate phosphoproteins.

Protein accession	Protein gene	Peptide modified sequence	Infected/NC peak ratio	Infected/NCP value	Infection/NC peak ratio (LPST)
F1RKM0	LMNB1	AGGPTT(ph)PLSPTR	2.62	9.37E−03	2.26
F6Q1M2	ASNS	WIS(ph)ASDPSAR	3.94	1.09E−04	4.46
Q6DUB7	STMN1	ASGQAFELILS(ph)PR	0.62	5.90E−03	0.32
D3K5N7	ELAVL1	NVALLSQLYHS(ph)PAR	0.37	6.12E−05	0.39
M3V846	RAF1	SAS(ph)EPSLHR	0.92	7.67E−02	2.21
A0A287A5B5	SRRM2	RVPS(ph)PAPAPK	1.15	3.88E−01	3.09
A0A287A5B5	SRRM2	QPGS(ph)PYEDKGK	2.22	1.64E−03	2.55
A0A287A5B5	SRRM2	TPAAAAAMNLAS(ph)PR	0.87	1.85E−01	0.75
A0A287A5B5	SRRM2	ILPQT(ph)PRPR	0.89	5.06E−01	0.77
A0A287A5B5	SRRM2	THSDSS(ph)PYPALDSK	0.83	4.23E−01	0.78
A0A287A5B5	SRRM2	NSGPVAEMSTEFS(ph)PEGK	0.90	1.35E−02	2.13
A0A287A5B5	SRRM2	AKPQTPPGHNLPESKS(ph)PCSQEK	1.33	2.37E−01	0.76
A0A287AEF3	BAD	SLS(ph)APPILWAAQR	1.68	1.64E−02	1.59
F1SSC2	CDK13	IEHAPS(ph)PSSSGTLK	0.43	1.01E−03	0.45
A0A287B726	IRS2	SKS(ph)QSSGSSATHPISVPGAR	0.72	5.74E−02	0.67
A0A287B726	IRS2	RHNS(ph)ASVENVSLR	0.61	5.19E−03	0.67
F1RS45	TOP2B	KAS(ph)GSENEGDYNPGR	0.86	3.31E−02	2.00
A0A2C9F3G4	STAT3	FICVTPTTCSNTIDLPMS(ph)PR	0.41	2.16E−04	0.50
I3LLX2	PIK3C2A	SKS(ph)AEVTSLSGGGGDASK	1.32	4.34E−02	2.12
F1S9K5	EPRS	KDASKT(ph)PESGLSPGGAGEGPGPK	0.73	4.32E−02	0.60
F1SUG7	DDX21	AEEMEEVIS(ph)PK	0.50	1.04E−06	0.61
F1SBP3	DDX20	LAAYHDS(ph)PEIQVK	0.44	1.66E−04	0.50
F1RST0	HSPH1	IES(ph)PKVER	1.00	9.88E−01	1.89
A0A286ZUQ1	ITPR1	RDS(ph)VLAASR	0.64	2.83E−02	1.02
Q6DUB7	STMN1	RAS(ph)GQAFELILSPR	1.09	3.29E−01	1.07
I3L6D8	EIF4EBP1	NSPVTKT(ph)PPR	0.74	1.82E−02	0.85
Q863I2	OXSR1	AAISQLRS(ph)PR	0.63	6.48E−02	0.66
F1RQS5	CDC5L	GGLNTPLHESDFSGVT(ph)PQR	0.64	8.41E−04	0.64
F1RJR9	PBK	GLSHS(ph)PWAVK	0.22	2.94E−05	0.41
I3LLX2	PIK3C2A	ATSSNLQVS(ph)PK	1.17	2.81E−01	3.02

The Student’s *t*-test examined whether phosphosites were differentially modified between the NC and infected groups. Quantifications of a given phosphosite in at least two out of three replicates were used for Student’s *t*-test. While *p* < 0.05 and fold change > 1.2 were used to identify differentially modified phosphosites.

As shown in Table 1, ASNS was the protein with the most upregulated phosphosites; the ratio of the peak area of the infected vs. the NC groups was 3.94. ASNS is an asparagine synthetase involved in the first step of the subpathway that synthesizes L-asparagine from L-aspartate, consistent with the GO annotation results of the phosphoproteomics analysis. ELAVL1, DDX21, and DDX20 are involved in the spliceosome pathway and RNA binding molecular functions, and their peak area ratios were 0.37, 0.50, and 0.44. Five of seven differentially regulated phosphosites were consistent with the results of phosphoproteomics, and the peak ratio of significant change phosphosite was 2.22. PBK and CDK13 are associated with the cell cycle, and their peak ratios were 0.22 and 0.43, respectively. BAD is a Bcl2-associated agonist that promotes cells death, and S140 phosphorylation was upregulated in this protein following SVA infection by 1.68-fold relative to controls. STAT3 is a signal transducer and transcription activator that mediates cellular responses to interleukins and other growth factors (Niu et al., 2019). According to the PRM analysis, the phosphorylation at S727 of STAT3 (STAT3-S727) was downregulated, with a peak area ratio of 0.41.

**Figure 8:**
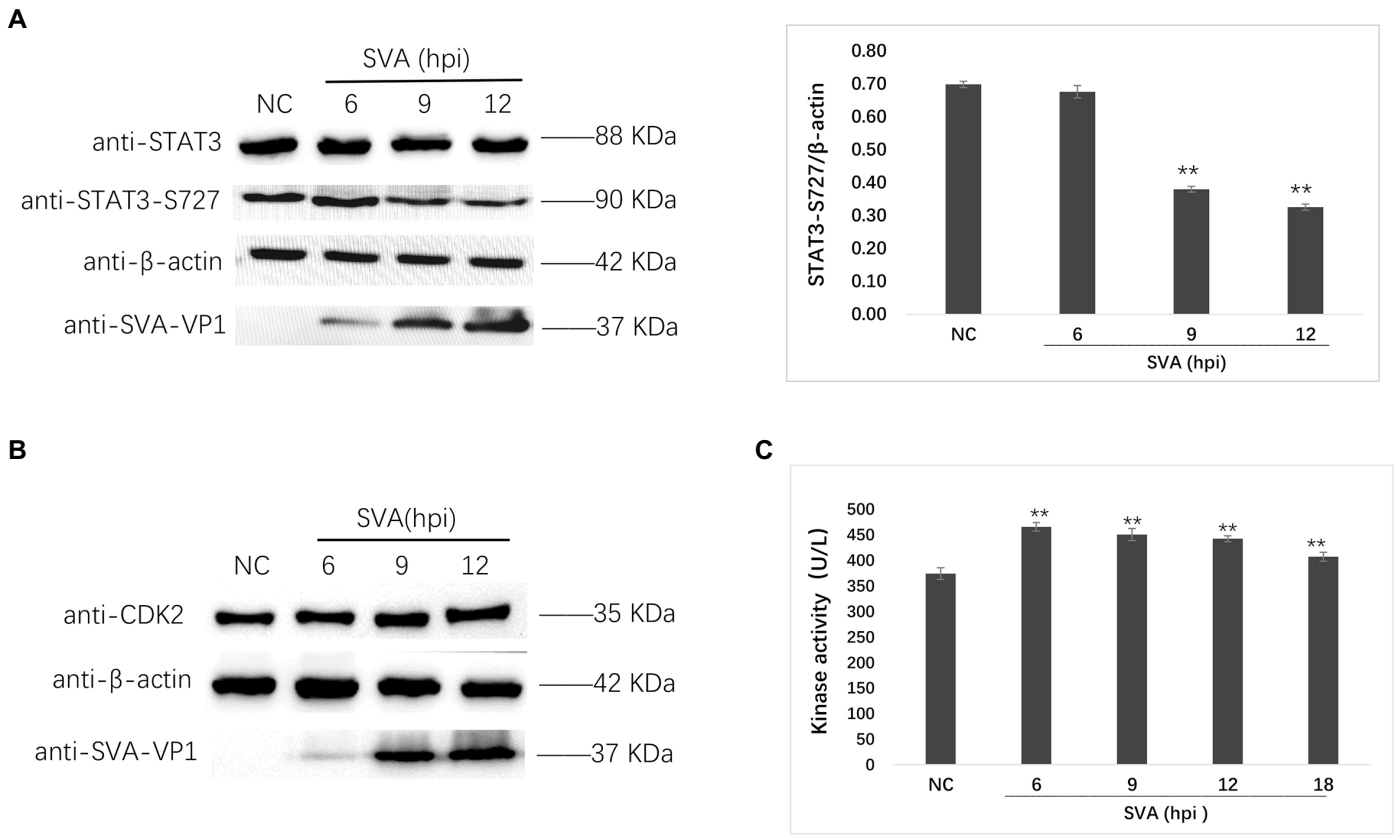
Verification of the phosphoproteomics results by Western blot and kinase activity detection. **(A)** Western blot detecting the expression of STAT3 and the phosphorylation of STAT3-S727 at 6, 9, and 12 hpi of SVA infection. The relative expression of STAT3-S727 was determined by comparing the STAT3-S727/β-actin area value. **(B)** The expression of CDK2 at 6, 9, and 12 hpi of SVA infection. **(C)** The kinase activity of CDK2 at 6, 9, 12, and 18 hpi of SVA infection. The statistical significance is shown by ** for *p* < 0.01.

Subsequently, we verified the phosphorylation at S727 of STAT3 (STAT3-S727) and the expression of CDK2 by western blot (Figures 8A,B). The phosphorylation of STAT3-S727 was downregulated after SVA infection, and the expression level of CDK2 remained unchanged, consistent with the previous results. Furthermore, the CDK2 kinase activity detection results were consistent with the kinase activity prediction (Figure 8C).

## Discussion

Senecavirus A exhibits multi-organ tropism; virus RNA has been detected in epithelial, tongue, gingiva, myocardium, lung, renal pelvis, liver, urinary bladder, brain, and small intestine tissues (Leme et al., 2016). The symptoms of SVA infection are similar to those of foot-and-mouth disease, especially blistering and ulceration of the rhinoscopy and hoof crown, which can lead to lameness. Piglets are susceptible, and severe infections can result in death. Numerous proteomics studies on picornavirus infections have been reported, especially FMDV (Ye et al., 2013; Guo et al., 2015).

Phosphoproteomics analysis of virus infection of host cells has been applied to influenza virus (Dapat et al., 2014), Sendai virus (Zhu et al., 2017a), porcine reproductive and respiratory syndrome virus (PRRSV; Luo et al., 2014a), and Gamma-herpesvirus (Stahl et al., 2013), among others. Phosphorylation is one of the most widespread PTMs, and it regulates numerous biological processes, including cell proliferation, growth, survival, and apoptosis (Stahl et al., 2013). However, quantitative phosphoproteomics profiling of SVA infection has not been performed previously. The present study used 4D label-free quantitative and phosphopeptide enrichment technology coupled with high-resolution LC-MS/MS analysis to establish a global map of differentially regulated protein phosphorylation during SVA infection.

Proteomics analysis of the same samples was initially conducted to exclude the impact of protein abundance. Additionally, proteomics and phosphoproteomics analyses were conducted using biological triplicates for each sample to improve the accuracy of the results. Based on the phosphoproteomics analysis, 40 phosphosites of 27 proteins were selected to validate the phosphoproteomics results by PRM analysis, and nine sites on nine proteins were selected for further investigation.

SRRM2 is a spliceosome protein involved in splicing pre-mRNA, and most mammalian pre-mRNAs are subjected to alternative splicing (AS; Ashraf et al., 2019), which is important for RNA maturation. In recent years, alteration of cellular splicing has been observed in many viral infections, which may result from the virus manipulating host cell splicing, virus-induced immune responses, or cellular damage (Prescott et al., 2014; Dhillon et al., 2018; Thompson et al., 2018). Picornaviruses such as FMDV can also manipulate the host splicing machinery (Lawrence et al., 2012), consistent with this study that the host splicing machinery may play a role in SVA infection. The previous studies on SRRM2 showed that it could interact with some viral proteins, increase nuclear permeability upon viral infection, and undergo differential phosphorylation following infection with HIV, leading to alteration of the host splicing machinery (Wojcechowskyj et al., 2013; Prescott et al., 2014). Further study is needed to confirm the function of SRRM2 in SVA infection of host cells. Like SRRM2, the RS protein and kinase CDK13 phosphorylates splicing factors such as ASF/SF2, thereby regulating HIV-1 mRNA splicing (Berro et al., 2008). CDK13 is an ATP-dependent serine-threonine protein kinase that regulates cell cycle progression. Its N-terminal domain contains RS motifs, mainly found in splicing regulators (Malumbres, 2014). CDK13 regulates splicing by altering the phosphorylation status and activity of splicing factors (Even et al., 2006). However, the relevance of CDK13 phosphorylation to SVA infection has not been discussed previously.

Another kinase, PBK, also known as T-lymphokine-activated killer cell originated protein kinase (TOPK), is induced by interleukin-2 (Abe et al., 2000). Interestingly, PBK is overexpressed in various cancer tissues (Luo et al., 2014b). Phosphorylation of PBK T9 and T198 is vital for its kinase activity and biological functions. In the current study, phosphorylation of PBK S59 was downregulated following SVA infection, and the significance should be further explored in the future.

ELAVL1 is an RNA-binding protein that binds to the 3′-untranslated region of mRNAs to stabilize them (Chen et al., 2019), and interferon-β mRNA and IL3 can affect the host immune response (Takeuchi, 2015). CDK1 phosphorylates ELAVL1 S202 and regulates its subcellular distribution during G2, ultimately inhibiting apoptosis (Kim et al., 2008). BAD, another apoptosis-associated protein and only pro-apoptosis member of the Blc-2 family, is regulated by rapid changes in phosphorylation of its Bcl-2 homology 3 (BH3) domain, which modulates its PPI and subcellular localization (Hsu et al., 1997; Datta et al., 2000). Phosphorylation of BAD at serine residues inhibits apoptotic activity. Survival factors such as IL-3 can activate intercellular signaling pathways, enabling phosphorylated BAD to bind to 14-3-3 proteins rather than Bcl-2 and Bcl-xL (He and Karin, 2011). As mentioned above, STAT3 is activated and translocated to the nucleus to promote target gene transcription. Phosphorylation of STAT3 at T705 (pTyrSTAT3) and S727 (pSerSTAT3) is required for full STAT3 transcriptional activity. pTyrSTAT3 causes STAT3 dimerization and translocation to the nucleus (Dziennis and Alkayed, 2008). pSerSTAT3 displays increased transcription activity independent of pTyrSTAT3. Inhibiting pSerSTAT3 induces thymocytes apoptosis, and pSerSTAT3 is critical for neuronal survival (Shen et al., 2004). Consistent with the KEGG pathway result, the differentially regulated phosphoproteins in the apoptosis pathway changed significantly, and they may play a role in SVA infection.

Other phosphorylated proteins were identified, including ASNS, DDX 20, and DDX21. Phosphorylation of ASNS and its significance in the interaction between viruses and host cells have not been investigated. Previous studies showed that ASNS is involved in oncogenesis in some cancer, in which it induces cell cycle arrest (Yang et al., 2014; Li et al., 2016). DDX20 (Gemin3 or DP103), an RNA helicase and member of the DEAD-box family, is a ubiquitously expressed 103 kDa phosphoprotein with RNA-dependent ATPase activity (Grundhoff et al., 1999). DDX20 is involved in the assembly and nuclear regeneration of snRNPs and spliceosomes. Epstein–Barr virus nuclear antigen 2 targets spliceosomes by binding to DDX20, resulting in the activation of the RNA-polymerase II transcription complex and initiation of viral replication (Voss et al., 2001). Thus, DDX20 may perform the same function described previously in Epstein–Barr virus infection. DDX20, like DDX21, is an RNA helicase linked to RNA biogenesis, transcription, and regulation of anti-viral host innate immunity (Chen et al., 2020). DDX21 can diminish influenza A virus protein expression by inhibiting RNA polymerase, which may be relevant to SVA infection. However, phosphorylation of DDX20 and DDX21 and its relevance to SVA infection has not been investigated.

Although our study focused on IBRS-2 cells infected with SVA, most of the sequences and functions of the identified proteins are highly conserved. Thus, the results are likely to apply to phosphorylation studies in other mammalian cells in response to infection with other similar viruses.

## Conclusion

Our phosphoproteomics approach identified proteins with differentially regulated phosphosites following SVA infection, and PRM validated the phosphoproteomics results. Kinase analysis predicted the regulatory relationships between kinases and the identified proteins (substrates), as well as kinases activities. The results provide comprehensive information on the potential roles of these phosphorylated proteins and their kinases in controlling cellular activities. The study provides a valuable reference for further studies on the pathogenesis and host cell responses of SVA infection.

## Data Availability Statement

The datasets presented in this study can be found in online repositories. The names of the repository/repositories and accession number(s) can be found at: https://www.ebi.ac.uk/pride/archive/, PXD026655; PXD022444.

## Ethics Statement

Ethical review and approval was not required for the animal study because without any animals or human experiments.

## Author Contributions

JLi and ZZ performed the experiments and analyzed the data. JLv and ZM prepared the figures and Supplementary Material. LP and ZZ designed the experiments and acquired the funding. JLi wrote the manuscript. YZ supervised the project. All authors contributed to the article and approved the submitted version.

## Funding

This work was supported by grants from the Natural Science Foundation of Gansu Province (Grant No. 20JR5RA584) and the China Agriculture Research System of MOF and MARA.

## Conflict of Interest

The authors declare that the research was conducted in the absence of any commercial or financial relationships that could be construed as a potential conflict of interest.

## Publisher’s Note

All claims expressed in this article are solely those of the authors and do not necessarily represent those of their affiliated organizations, or those of the publisher, the editors and the reviewers. Any product that may be evaluated in this article, or claim that may be made by its manufacturer, is not guaranteed or endorsed by the publisher.

## Supplementary Material

Supplementary Table S1 Protein abundance proteomic data for IBRS-2 cells infected with SVA. A full list of protein abundance measurements following SVA infection in IBRS-2 cells is included (Quant tab).

Supplementary Table S2 Phosphorylation and abundance data related to Figures 2B,C. Phosphoproteomic data are for IBRS-2 cells infected with SVA. A full list of unfiltered phosphosites detected following SVA infection is included (PhosphoData unfiltered tab), along with a filtered list of all detected phosphosites collapsed into single-site measurements (PhosphoData Filtered tab).

Supplementary Table S3 Predicted kinase activities related to Figures 7A–C. The full list of predicted kinase activities for IBRS-2 cells in NC and infected groups is included (NC and infected tabs, respectively).

Supplementary Table S4 Phosphopeptide abundance data related to Table 1. The full list of results for PRM validation of filtered phosphosites in IBRS-2 cells following SVA infection is included (PhosphoPRM tab).
